# Determinants of early neonatal mortality (hospital based retrospective cohort study in Somali region of Ethiopia)

**DOI:** 10.1038/s41598-023-28357-x

**Published:** 2023-01-20

**Authors:** Ahmed Tahir Ahmed, Abdifatah Elmi Farah, Hussein Nooh Ali, Muse Obsiye Ibrahim

**Affiliations:** 1 Public Health Department, College of Medicine and Health Science, Jigjiga University, P.O.Box: 1020, Jigjiga, Ethiopia; 2 Pediatric and child health Specialty Department, College of Medicine and Health Science, Jigjiga University, Jigjiga, Ethiopia; 3 Health Sector, Jigjiga field office, UNICEF, Jigjiga, Ethiopia

**Keywords:** Health care, Paediatrics, Neonatology

## Abstract

Early neonatal mortality occurs when a newborn dies within the first seven days of life. Despite interventions, neonatal mortality remains public health problem over time in Ethiopia (33 per 1000 live births). Determinants varies on level of neonatal mortality. The study's goal was to determine magnitude of early newborn death, as well as its determinants and causes in Newborn Intensive Care Unit of Referral hospital in Ethiopia's Somali region. Health facility based retrospective study review was conducted between May 2019 to May 2021 in Shiek Hassan Yabare Referral Hospital of Jigjiga University of Ethiopia. All neonates admitted at neonatal intensive care unit (NICU) with complete data and get registered using the new NICU registration book from May 2019 to May 2021 were included. Kobo toolkit was used for data collection and analyzed in SPSS 20. The magnitude of early neonatal mortality rate was defined as death between 0 and 7 days. Logistic regression model was used to estimate maternal and neonatal characteristics as a determinant variables on neonatal mortality. The statistical significance was considered at P-value < 0.05. The magnitude of early neonatal mortality rate of Ethiopia’s Somali region is estimated to be 130 per 1000 live births—that is say 130 newborn couldn’t celebrate their seventh day in every 1000 live births. Hypothermia, prematurity, maternal death at birth and shorter length of stay in NICU were increasing the chance of neonatal mortality at early stage while neonatal resuscitation had shown protective effect against neonatal mortality. Similarly birth asphyxia, preterm, sepsis, and congenital abnormalities were major causes of admission and death in the NICU. The magnitude of early neonatal mortality is considerable and most of the determinants are preventable. Enhancing quality of intra-partum and NICU care including infection prevention, managing hypothermia and neonatal resuscitation as per the national standard within the first golden hour is key.

## Introduction

Early neonatal mortality occurs when a newborn dies within the first seven days of life^1^.

Neonatal mortality continues to be the highest (17%) among children under the age of five worldwide, with Sub-Saharan Africa accounting for the largest share^2^. Low-income countries continue to have higher rates of neonatal death^3,4^.

Despite interventions, newborn mortality remains public health problem in Ethiopia, it has come down from 49 to 30 and only now is 33 per 1000 live births^5^. However, a comprehensive study and meta-analysis found that early newborn mortality is reducing in Ethiopia, despite the high rate of early neonatal mortality (29.5 per 1000)^6^.

Furthermore, even newborns admitted to sub-Saharan nations' neonatal intensive care units (NICU)^7–10^, including Ethiopia, have a greater rate of early neonatal mortality. The causes of newborn mortality are numerous and vary depending on the situation. Prematurity and congenital anomalies^1^ and low APGAR score^11^ are the leading causes of early newborn mortality in the developed world, whereas very low birth weight^9,12^ sepsis^13^ and asphyxia^7,9,10,12,14^ are the leading causes in the low-income world, according to evidence from systematic reviews.

In low-income countries such as Ethiopia, early newborn mortality may also be caused by insufficient access to care^7,15,16^, home birth^16^, and issues with NICU care quality^4,8^.Prematurity-related problems and late breast-feeding initiation are also causes of infant death in low-income and resource-constrained countries^7,13,14,17^.

Furthermore, maternal illness and death, as well as a poor Apgar score, are two additional causes of neonatal mortality^7,12,13,18^. Maternal child spacing, on the other hand, protects against it^6^. Finally, duration of stay is another driver in Ethiopia, particularly in pastoralist communities, in addition to the other primary causes of newborn mortality in low-income countries like Ethiopia^8,19^.

The magnitude of early neonatal mortality in Ethiopia is poorly understood, and its determinants are varies in different context. The study's goal was to determine the magnitude of early newborn death, as well as its determinants and causes, in NICUs of health facilities in Ethiopia's Somali region.

## Methods

All methods were carried out in accordance with relevant guidelines and regulations.

### Study setting and period

Health facility based retrospective study review was conducted between May 2019 to May 2021 in Shiek Hassan Yabare Referral Hospital (SHYRH) of Jigjiga University, Ethiopia. SHYRH began delivering services in 2017 and it serves about 5 million people in Somali region of Ethiopia. The hospital has 400 beds and serves 86,000 outpatients annually. It is the largest hospital with a number of specialties including neonatal intensive care unit (NICU) in Somali region, Ethiopia. The number of average deliveries per month is 400.

### Eligibility criteria

Neonates died or survived were reviewed among neonates admitted in NICU of SHYRH from May 2019 to May 2021. All neonates admitted at NICU and get registered using the new NICU registration book from May 2019 to May 2021 were included. Early neonatal death was considered a newborn death within the first week of life (between 0 to 7 days) while in NICU^1^. Live births were evaluated up to the 7th day after birth. Admitted neonates with incomplete data were excluded.

### Sample size

Between April and May of 2021, an exhaustive review of the NICU registration book from May 2019 to May 2021 was conducted, with a total of 765 eligible neonates (99 deaths and 666 survivors) included in the study.

### Data collection methods

The data was collected using checklist extracted from the new NICU registration book as it is comprehensive and filled properly. Two neonatal Nurse by profession have collected the data using Kobo toolbox software to prevent errors happen during data collection. Data collectors were sending the collected data per day to the server and getting on spot feedback from the supervisors.

The outcome variable of the study was early neonatal death at NICU of SHYRH while maternal and neonatal characteristics were as an independent variables. Mode of delivery, place of delivery, HIV test, Syphilis test, Hepatitis B test and maternal status were considered as maternal characteristics while sex, birth weight, temperature, APGAR score, length of stay, resuscitation, prematurity, sepsis, respiratory distress syndrome, Asphyxia, congenital malformation and Kangaroo mother care(KMC) were considered as neonatal characteristics.

#### Operational definition

The magnitude of early neonatal death is the proportion of neonatal death at hospital within the first 7 days of life among total live births admitted to the NICU.

Causes of neonatal mortality is any medical diagnosis that pediatricians noted after doing the required laboratory analysis and documented at patient chart as causes of neonatal death.

Kangaroo mother care (KMC) is a skin to skin contact between a mother and her newborn.

Birth weight is classified as low birth weight when it is less than 2500 g, and Overweight when it is ≥ 4000 g according to WHO.

Sepsis: any infection that was diagnosed based on clinically or laboratory investigation by health professionals in charge at NICU during the neonate's admission and is recorded on the patient chart.

Hypothermia: is any low body temperature measurement below 36.5 °C that was identified and documented on charts by physician during the admission of newborns at NICU.

Prematurity: Any live-born neonate who is delivered before 37 full weeks according to health professionals in charge at the NICU during admission of neonate and recorded at patient chart.

Birth asphyxia: when a newborn's Apgar score is below 6 in the fifth minute of life, or if the child don’t not cry right after birth, experienced respiratory difficulty, floppiness, loss of consciousness, the presence of convulsions, and loss of neonatal reflexes.

The Apgar score is a quick way for health professionals to evaluate the health of all newborns at 1 and 5 min after birth and in response to resuscitation.

Congenital malformations: structural or functional birth defects that occur during intrauterine life.

### Data management and analysis

For data entry and collection, the Kobo toolkit was used. The data was cleaned with the Kobo toolkit before being analyzed with SPSS version 20. To find factors of early newborn death, researchers used bivariate and multivariable binary logistic regression. In multivariate analysis, variables having a P-value less than 0.2 in bivariate analysis, as well as other relevant determinants of early newborn mortality, were evaluated. In multivariate binary logistic regression analysis, factors having a P-value less than 0.05 were determined to have a significant relationship with the outcome variable. The odds ratio with 95 percent confidence interval was used to estimate the strength of the association. Stata version 16 with command of Prop 765 (size) 99 (outcome), exact (95% CI) was used to estimate the magnitude of early neonatal mortality rate.

Furthermore, to have a better knowledge of neonatal death, a frequency distribution table was employed to discover the causes of early neonatal mortality. The causes of neonatal death with highest proportions in the frequency distribution was reported as the top causes of neonatal mortality.

### Ethical consideration

The study was ethically approved by ethical committee of college of medicine and health science of Jigjiga University, Ethiopia. Since data was collected from NICU registration book of SHYRH, an official consent was obtained from Hospital administration.

## Results

### Newborn characteristics at NICU

A total of eligible 765 neonates with complete data were reviewed and included in the study to estimate the rate of early neonatal mortality in Ethiopia's Somali region based on neonates admitted to the Shiek Hassen Referral Hospital's NICU. Among 765(between 0 and 7 days) reviewed, 99 died early and 666 survived. Higher proportion 41/99 (41.4%) of small babies (below 2000 g) died relative to 184/666 (27.6%) survived. similarly, 88/99 (88.9%) and 72/99 (72.7%) of the 99 neonates who died early were hypothermic and not resuscitated respectively. Regarding the first minute APGAR scores, mortality were higher among neonates with lower score 88/99 (88.9%) compared to those with higher score 11/99 (11.1%) which is expected.

Survival rate was lower among neonates admitted with premature 63/666 (9.5%), asphyxia 143/666 (21.5%) and congenital anomalies 65/666 (9.8%) compared to counterparts. Lastly, early neonatal mortality was lower 13/99 (13.1%) among neonates received KMC compared to those not received 86/99 (86.9%) (Table 1). In addition, to assess the level of KMC utilization of the study Hospital, cross tabulation of Hypothermia and KMC was done and the result shows that a total of 504 hypothermic neonates, only 64 (12.3%) have received KMC which is very low.

**Table 1:** Characteristics of NICU admitted neonates at Referral Hospital, Jigjiga from May 2019–2021.

Newborn characteristics		Discharge status	
		Death (%), n = 99	Survived (%), n = 666
Sex of the baby	Male	54 (54.5%)	363 (54.5%)
	Female	45 (45.5%)	303 (45.5%)
Birth Weight	< 2000 g (small baby)	41 (41.4%)	184 (27.6%)
	2000–2499.99 g (LBW)	20 (20.2%)	74 (11.1%)
	≥ 4000 g (large baby)	1 (1.0%)	22 (3.3%)
	2500–3999.99 g (normal)	37 (37.4%)	386 (58.0%)
Temperature	Normal (36.5–37.5)	10 (10.1%)	223 (33.5%)
	Low temperature (< 36.5)	88 (88.9%)	416 (62.5%)
	High temperature (> 37.5)	1 (1.0%)	27 (4.1%)
APGAR Score 5th min	1–6	58 (58.6%)	385 (57.8%)
	7–9	41 (41.4%)	281 (42.2%)
APGAR Score 1th min	0–6	88 (88.9%)	547 (82.1%)
	7–9	11 (11.1%)	119 (17.9%)
Length of stay	< 1 day	4 (4.0%)	12 (1.8%)
	2 to < 7 days	94 (94.9%)	498 (74.8%)
	7 days plus	1 (1.0%)	156 (23.4%)
Resuscitation	No	72 (72.7%)	593 (89.0%)
	Yes	27 (27.3%)	73 (11.0%)
Prematurity	No	67 (67.7%)	603 (90.5%)
	Yes	32 (32.3%)	63 (9.5%)
Sepsis	No	55 (55.6%)	310 (46.5%)
	Yes	44 (44.4%)	356 (53.5%)
Respiratory distress syndrome (RDS)	No	34 (34.3%)	272 (40.8%)
	Yes	65 (65.7%)	394 (59.2%)
Perinatal asphyxia	No	67 (67.7%)	523 (78.5%)
	Yes	32 (32.3%)	143 (21.5%)
Congenital malformation	No	89 (89.9%)	601 (90.2%)
	Yes	10 (10.1%)	65 (9.8%)
KMC	No	86 (86.9%)	604 (90.7%)
	Yes	13 (13.1%)	62 (9.3%)

### Magnitude of early neonatal mortality

The magnitude of early neonatal mortality rate of Ethiopia’s Somali region was estimated to be 130; 95% CI (106–154) per 1000 live births. That is 130 newborn couldn’t celebrate its sevens day in every 1000 live births. It was estimated from 765 live births admitted throughout the study period, with 99 deaths occurring during the first 7 days.

### Maternal characteristics of neonates admitted to NICU of referral hospital

Many maternal characteristics were taken into account as determinants. Survival rate was lower among neonates born at home, by instrumental delivery and their mother died during childbirth relative to counterparts (Table 2).

**Table 2:** Maternal characteristics of neonates admitted to NICU of Shiek–Hassen Referral Hospital from May 2019–May 2021.

Maternal characteristics		Discharge status	
		Death (%), n = 99	Survived (%), n = 666
Mode of delivery	Cesarean section (CS)	23 (23.2%)	182 (27.3%)
	Instrumental	1 (1.0%)	22 (3.3%)
	Spontaneous	75 (75.8%)	462 (69.4%)
Place of delivery	Home delivery	3 (3.0%)	20 (3.0%)
	Referred from other	17 (17.2%)	158 (23.7%)
	Same facility	79 (79.8%)	488 (73.3%)
HIV test	Negative	98 (99.0%)	661 (99.2%)
	Positive	1 (1%)	5 (0.8%)
Hepatitis B test	Negative	96 (97.0%)	649 (97.4%)
	Positive	3 (3.0%)	17 (2.6%)
Syphilis test	Negative	99 (100.0%)	664 (99.7%)
	Positive	0 (0.0%)	2 (0.3%)
Maternal status	Alive	77 (77.8%)	661 (99.2%)
	Dead	22 (22.2%)	5 (0.8%)

### Top causes of early neonatal mortality at NICU of Shiek Hassen referral hospital, Somali, Ethiopia.

Prenatal Asphyxia was the major cause of early neonatal mortality, followed by prematurity, with 32 (32.3%) and 31 (31.3%) deaths, respectively. Sepsis and congenital anomalies were the third and fourth causes of early neonatal mortality, respectively, 24 (24.2%) and 8 (8.1%) (Table 3).

**Table 3:** Top causes of early neonatal mortality at NICU, Referral Hospital from May 2019 to May 2021.

Ranks	Causes of early neonatal mortality	Frequency (%)
1	Prenatal asphyxia	32 (32.3)
2	Prematurity	31 (31.3)
3	Sepsis	24 (24.2)
4	Congenital malformation	8 (8.1)
5	Others	4 (4.0)

### Determinants of early neonatal mortality at NICU of Shiek Hassen Referral hospital

All newborn and maternal characteristics with a P-value less than 0.2 were considered in multivariable analysis. However; maternal mortality, preterm, newborn resuscitation, hyperthermia, and newborn shorter length of stay in NICU were revealed to be determinants of early neonatal mortality. When compared to those whose moms were alive, newborns whose mothers died during childbirth were considerably more likely to die early at neonatal age [adjusted odd ratio (AOR) 29.3; 95% CI 9.3, 92.1]. Similarly, the neonatal mortality were highest within the first 24 h after birth significantly AOR 72.5; 95% CI (6.3, 834.2) compared to deaths beyond the first 24 h till 7 days of live. Furthermore, compared to term born newborns and neonate with normal temperature, premature and hypothermic babies were almost four times more likely to die early at neonatal age (AOR 3.6; 95% CI (1.7, 8) and AOR 3.8; 95% CI (1.8, 8.1) respectively. Newborn resuscitation, on the other hand, has a protective effect and reduces early newborn death by 70% AOR 0.3; (0.2, 0.8) (Table 4).

**Table 4:** Multivariable logistic regression analysis of maternal and newborn characteristics by early neonatal mortality, NICU of Referral hospital from May 2019 to May 2021.

Maternal and newborn characteristics		Discharge status		95% CI COR	AOR	P-value
		Dead (%)	Survived (%)			
Maternal status	Alive	77 (77.8%)	661 (99.2%)	1	1	
	Dead	22 (22.2%)	5 (0.8%)	37.7 (13.9, 102.6)	29.3 (9.3, 92.1)**	< 0.001
Place of delivery	Same facility	79 (79.8%)	488 (73.3%)	1	1	
	Home delivery	3 (3.0%)	20 (3.0%)	1.1 (0.3, 3.7)	1 (0.2, 4.9)	0.978
	Referred from other	17 (17.2%)	158 (23.7%)	1.5 (0.9, 2.6)	0.8 (0.2, 4.2)	0.814
Prematurity	No	67 (67.7%)	603 (90.5%)	1	1	
	Yes	32 (32.3%)	63 (9.5%)	4.6 (2.8, 7.5)	3.6 (1.7, 8)**	0.001
Sepsis	No	55 (55.6%)	310 (46.5%)	1	1	
	Yes	44 (44.4%)	356 (53.5%)	0.7 (0.4, 1.1)	1.3 (0.7, 2.3)	0.314
Asphyxia	No	67 (67.7%)	523 (78.5%)	1	1	
	Yes	32 (32.3%)	143 (21.5%)	1.7 (1.1, 2.7)	2.2 (0.9, 4.9)	0.054
Resuscitated	No	72 (72.7%)	593 (89.0%)	1	1	
	Yes	27 (27.3%)	73 (11.0%)	0.3 (0.2, 0.5)	0.3 (0.2, 0.8)**	0.012
Birth weight	2500–3999.99 g (normal)	37 (37.4%)	386 (58.0%)	1	1	
	< 2000 g (small baby)	41 (41.4%)	184 (27.6%)	2.3 (1.4,3.7)	1.9 (0.9, 4.0)	0.099
	2000–2499.99 g (LBW)	20 (20.2%)	74 (11.1%)	4.9 (0.6,37.4)	2.5 (0.3, 21.5)	0.398
	≥ 4000 g (large baby)	1 (1.0%)	22 (3.3%)	0.8 (0.4,1.5)	0.8 (0.3, 1.7)	0.501
Temperature	Normal (36.5–37.5)	10 (10.1%)	223 (33.5%)	1	1	
	Low temperature (< 36.5)	88 (88.9%)	416 (62.5%)	4.7 (2.4, 9.3)	3.8 (1.8, 8.1)**	0.001
	High temperature (> 37.5)	1 (1.0%)	27 (4.1%)	5.7 (0.7,42.6)	3.1 (0.4, 24.7)	0.290
Length of stay	7 days plus	1 (1.0%)	156 (23.4%)	1	1	
	< 1 day	4 (4.0%)	12 (1.8%)	52 (5.4, 502.6)	72.5 (6.3, 834.2)**	0.001
	2 to < 7 days	94 (94.9%)	498 (74.8%)	1.7 (0.5, 5.6)	2 (0.5, 8.7)	0.341

*Significant at P-value < 0.05 **Significant at P-value < 0.01.

## Discussion

In present study, the magnitude of institutional early neonatal mortality rate was 130; 95% CI (106–154) per 1000 live births in a NICU of SHYRH of Jigjiga University in Ethiopia's Somali region. Maternal mortality, prematurity, newborn resuscitation, hypothermia, and newborn short duration (< 24 h) in NICU are the determinants of early neonatal mortality. The magnitude of early neonatal mortality rate of the current is very high and mainly defines the quality of obstetric and NICU care in the study facility. However, this has to be carefully interpreted as the neonatal death delivered in a facility, but who die outside the facility in the first 7 days of life is not captured and not included in the calculation of this death rate. The major causes of admission and death in the newborn Intensive Care Unit include birth asphyxia, prematurity, sepsis, and congenital abnormalities. This is in line with the findings of other investigations^10,17,20,21^.

The magnitude of early neonatal mortality rate of the current is very high and mainly defines the quality of obstetric and NICU care in the study facility. It is by far higher than a study in Afghanistan that showed 1.4 percent^22^, Northern Ethiopia (1.86%)^23^, Bukinafaso [2.6%]^24^, Nigeria (3.8%)^25^ and shaanxi province, China (7.9%)^26^. This might be due to different sample size and quality of obstetric and newborn care across context.

Prematurity was discovered to be a significant determinant of early newborn mortality. This is in line with the fact that preterm newborns have a greater mortality rate than term newborns^27^. Organ failure, neurodevelopmental and learning disabilities, vision problems, and long-term cardiovascular and non-communicable diseases are all risks for preterm babies^28,29^. The findings of this study are likewise consistent with those of Ethiopian and Sub-Saharan African studies^7,8,10,18^.

Another important determining factor that exhibited a protective effect against early neonatal mortality was neonatal resuscitation. This may be due to the fact that resuscitation is helping neonates with perinatal Asphyxia which is one of the top causes of neonatal mortality in the study setting. As studies in low income countries including systematic review suggested, appropriate help baby birth or resuscitation increases survival of first day of life but not all neonatal mortality^30–32^. This means in addition to resuscitation, other neonatal interventions are needed to prevent neonatal mortality^30^. It is also in line with findings of a Delphi panel of 18 experts, who indicated that urgent newborn facility-based resuscitation would avoid an additional 10% of preterm deaths, while community-based resuscitation would prevent an additional 20% of intrapartum-related and 5% of preterm fatalities^33^. Several other studies of neonatal resuscitation in low- and middle-income countries have shown that it has the potential to save newborn lives^34–36^.

Hypothermia (lower than normal body temperature) has also been linked to early newborn death. This is consistent with researches conducted in Ethiopia and elsewhere, which found that neonatal admission hypothermia dramatically increases the chance of death^37,38^. Hypothermia during NICU admission increased the risk of early newborn death, according to another study^39,40^. This could be linked to procedures such as delivering babies at < 25 °C (delivery room temperature), providing respiratory assistance with cold air during transfer to the NICU, and not using a cap on newborns, as well as unnecessary delays in skin-to-skin contact, preterm, and significant bacterial infection^40–42^.

Furthermore, the majority (442, or 87.7%) of hypothermic neonates admitted to the study hospital did not receive Kangroo mother care (KMC), which protects the newborn from infection, effectively treats hypothermia, improves gastrointestinal function and cardiorespiratory stability, and encourages breast feeding^43^ and thus reduces early neonatal mortality^44,45^.

The neonatal mortality were highest within the first 24 h after birth significantly AOR 72.5; 95% CI (6.3, 834.2) compared to deaths beyond 24 h. The risk of dying is highest in the first day of life in low income countries as systematic review on when do newborns die shows^46^. The finding is also consistent with researches that show majority of neonatal deaths occur in the first week of life, particularly in the first 24 h^7–9,47,48^. During the neonatal era, the risk of death is highest at the time of birth and gradually diminishes over the following days and weeks. Within the first 24 h of delivery, up to 36% of neonates die, and approximately 73% die within the first week of life^49^. Thus establishing NICU at Hospital is not enough but quality of care interventions given at birth and immediately after birth is very critical for neonatal survival^50^. This finding is also in line with research in Jimma of Ethiopia (< 7 days) and eastern Ethiopia (< 3 days) which found that shorter duration are associated with higher neonatal mortality^10,51^.

The other determinant of early newborn mortality discovered in this study was maternal death; newborns whose mothers died were more likely to die; the early post-natal period is a dangerous time for both mothers and their babies, and is strongly related to labor, intra-partum, and immediate new-born care practices. Studies have found a handful of maternal factors for early newborn death, such as hemorrhage and pregnancy-induced hypertension^7,52–56^. Furthermore, poor-quality care is responsible for 61% of neonatal deaths and half of maternal mortality^50^. This is because newborn care cannot be offered in isolation; it must be provided in conjunction with high-quality maternal care, which is also critical in saving lives. The health of mothers and their babies is intertwined, and providing appropriate interventions has the potential to save 71 percent of newborn fatalities, 33 percent of stillbirths, and 54 percent of maternal deaths if implemented fully^57^.

This study has the advantage of reviewing all newborns admitted to the hospital's NICU from 2019 to 2021, eliminating any potential sampling error. However, limitations of the study are related to the fact that facility-based studies do not reflect much of the neonatal deaths happening at community level and existence of poor-quality documentation for deaths.

## Conclusion

The magnitude of early neonatal mortality at Neonatal Intensive Care Unit is very high and this magnitude is generalizable only to health facilities in setting. The determinants are maternal and neonatal related factors which are preventable. Maternal mortality, prematurity, newborn resuscitation, hypothermia are determining the early neonatal mortality in the study setting. In addition, the risk of newborn dying is higher in the first day of life after birth in NICU. Enhancing quality of obstetric and essential care for newborn babies as per the national standard including prevention of infection and hypothermia within the first golden hour is supper important of the Hospital.

## Supplementary Information

Supplementary Information.

## Data Availability

The dataset is available at supplementary S1 file. SPSS dataset.

## Abbreviations

NICU: Neonatal intensive care unit
RDS: Respiratory Distress Syndrome
CS: Cesarean section
KMC: Kangaroo mother care
LBW: Low birth weight
SHYRH: Shiek Hassan Yabare Referral Hospital (SHYRH) of Jigjiga University
